# User Involvement in Myasthenia Gravis Research

**DOI:** 10.3389/fneur.2022.839769

**Published:** 2022-06-02

**Authors:** Nils Erik Gilhus, Sandra Iren Barkås Hovland

**Affiliations:** ^1^Department of Clinical Medicine, University of Bergen, Bergen, Norway; ^2^Department of Neurology, Haukeland University Hospital, Bergen, Norway; ^3^Norwegian Association for Muscle and Nerve Disorders, Oslo, Norway

**Keywords:** myasthenia gravis, user involvement, autoimmunity, patient representation, patient participation

## Introduction

Myasthenia gravis (MG) is an autoimmune disease with muscle weakness as the main manifestation (1). The disease is chronic, often with life-long symptoms. Most patients need daily immunosuppressive and cholinergic drug treatment (2). Although much is known about disease mechanisms, the cause of MG is unknown, and no curative treatment is available. MG is classified as a rare disease, with an annual incidence 10 per million in most populations, and a prevalence of 150–250 per million (3, 4).

The need for MG research is high. This is true for nearly all aspects of the disease. Genetic and environmental factors interact in causing MG. Most of the genetic predispositions remain unexplained, and we know even less about causative environmental factors. Although effective treatment is available, there are only a few placebo-controlled therapeutic trials. Studies comparing different treatment alternatives are lacking, and prospective, long-term follow-up studies are sparse. Research regarding burden of disease, quality of life, non-muscle symptoms, and the effect of supportive therapies and non-pharmacological interventions has emerged in recent years, but unbiased and well-conducted studies are only a few (5–7).

MG comprises a wide variation of phenotypes. Disease subgroups have been defined from age at symptom debut, generalization of symptoms, autoantibody profile and thymus pathology (8). Combinations of biomarker pattern and clinical manifestations will probably lead to further subgrouping of MG patients in the future and guide a more individually adapted treatment. In addition to phenotypic variation, there are important geographical differences. In China and Japan, there is a group of patients with MG onset in very early childhood (9–11). In Europe and North America, late onset MG is by far the most common type, in part due to demography. Availability of therapeutic and therapeutic alternatives as well as organization of MG care vary even more world-wide.

Patient involvement in health care is not only desirable but is a social, technical, and economic necessity (12). This includes treatment of MG. Patients are generally positive to take part as objects in research projects (13). In addition, they regard their active involvement as user representatives as important. MG research needs the input from patients who have experienced the various symptoms, examinations, and therapies, as well as the multiple consequences of having MG. MG patients know from experience the needs for a precise diagnosis and better treatment, for correct information and more knowledge. The linguistic shift from “patient” to “user” reflects a change in ideology (14). Our recent paper has illustrated the complex needs of MG patients (15). The patients themselves should be partners in the project to improve the present situation. Such user involvement should be adapted according to the phenotypic variation of MG. Some MG research questions are universal, whereas others are specific for children, pregnant women, the very old, immigrants, or other patient subgroups.

In this paper, our aim is to examine the relevant literature, make a narrative review, discuss the need of active involvement from MG patients in research projects relevant for this disease, and then conclude with several recommendations applicable in active MG research. User involvement should improve research relevance and quality, but also patient inclusion and continuation rates, project funding, dissemination of the results, and implementation of new knowledge into clinical practice.

## Patient Needs

Most MG patients do well and have a good prognosis (16). Expected life-length is nearly unaffected by the disease in well-developed countries (17, 18). Long-term studies typically report that a clear majority of MG patients are in full or partial remission, with no or only mild symptoms. In contrast, 10–20% of the patients have a disease that is difficult to treat and with a need for intensified treatment, whereas <5% have long-lasting severe MG (10, 16, 19). Many MG patients can function in daily life and partake in the ordinary labor market. However, while MG may be considered less severe than some other neurological and autoimmune conditions, most patients report reduced quality of life.

When questioning MG patients about specific complaints and limitations, it becomes clear that MG is a disease that has an impact on daily life and with a clear need for new and better treatments (5). A broad range of symptoms and deficits can be recorded as scores in MG-specific outcome forms or registered by specific questioning during ordinary consultations (20). In our recent article where we applied the MG patient perspective, we discussed patient needs in detail (15). The article was co-authored by MG experts and user representatives from three different countries.

Burden of MG disease is not clearly related to degree of muscle weakness (21). It depends in part on such phenotypic aspects as sex and age (5). Younger patients and females report more limitations and a poorer disease-specific quality of life. Quality of life does not seem to have improved for MG patients during the last decades, despite more effective immunosuppressive treatment (15). Nearly one third of MG patients answered “no” when asked if they were satisfied with their current MG status (22). In choosing optimal treatment, patients are interested in reports from other patients with the same diagnosis. Such patient experiences can be collected systematically (23).

To the patients it is the overall quality of life that is of greatest concern. Setting realistic expectations of the disease through systematically collected patient data may be beneficial both for them and their families. Patients are disappointed if it turns out that their disease is not as mild and easily managed as they had hoped for. Systematically collected patient experiences are useful not only to other patients but also to researchers and clinicians. Patient organizations typically have programs to get the newly diagnosed in contact with other patients to share experiences and ideas for managing their MG. User representatives could bring such records into research projects.

Muscle strength improvement is the major aim in MG treatment. For the patient, strength in some muscles is crucial, whereas weakness localized to other muscles has less impact. However, improved strength also in muscles not very important for patients' daily life can be crucial for the objective assessment of a new treatment. Fatigue is in some patients a major symptom (6). Patients feel weak and tired, often in most of the body, and this fatigue responds less well to immunosuppressive and anticholinesterase drugs than the muscle weakness. Physician-based and patient-focused assessments are both included in modern MG trials. Side-effects and worries about long-term consequences of the MG therapy are common. This includes infection risk and reduced vaccination response due to immunosuppression (24), but also cancer risk (25). Possible consequences of MG and MG therapy for pregnancy and the developing child mean that many young MG females postpone or abstain from becoming pregnant (26). Pain and depression are more common in MG patients than in controls. Comorbidities are frequent in MG, and especially in elderly patients (25). These comorbidities can be associated with their MG (other autoimmune disorders, thymoma, drug side effects), they can functionally interact with MG (lung disease, orthopedic disorders), or they can just add to the total burden of disease.

MG patients are eager to support research that in the future may improve their function and quality of life. Myasthenia Gravis Foundation of America (MGFA) and similar national MG patient organizations lists research support among their highest priorities (https://myasthenia.org/Research). Most fields of medical research are relevant in the patient perspective and may benefit from user participation. Diagnostic precision is important for both patient and neurologist and should include MG subgroup and phenotype (15). Patients know that research on disease mechanisms are necessary to improve treatment. MG causative factors, both environmental and genetic, might be preventable or possible to modify, and basic research is the way to reveal them. Treatment studies are most important in the patient perspective, not least prospective and controlled studies comparing alternative treatment protocols. MG patients are eager to contribute to research with the aim of defining optimal availability and organization of MG care. Resources and local priorities will influence MG treatment (27). Research regarding best organization should therefore be nationally or regionally adapted, and always with active user participation.

Studies evaluating to what degree diagnostic procedures and treatment of individual MG patients are consistent with generally accepted guidelines and recommendations are much needed (28). Such studies could unveil lack of knowledge, lack of availability and resources, compliance and organizational issues, but also a need for improved cost-benefit considerations in the recommendations. Active study participation from MG patients both in the planning and in the evaluation and dissemination phase should increase the scientific quality and the relevance for clinical practice.

## Patient Involvement

The aim for patient involvement in MG research is to improve quality, increase research output, increase relevance, support dissemination of results, and secure implementation in clinical practice. These are the main reasons why many institutions and funding sources demand user involvement in planned and ongoing medical research. User involvement in research is in addition justified from common ethical ideals. Individuals that are affected by the disease in focus should have the opportunity to influence activities so important for them (14). User involvement ensures that those who are affected can contribute with their knowledge and lived experience. User involvement is well established in most fields of society, including the clinical practice of hospitals and other health institutions. Medical research is such an important sector that broad involvement from the society is necessary. Patients and other users should get the chance to contribute. Their practical participation may be influenced by their MG symptoms such as diplopia (difficult to read) and fatigue.

In the planning phase of a new MG research project, patients can often give important input (29–32). Clinical relevance is an obvious aspect for them to discuss. They may also suggest additional approaches or new topics for research. Furthermore, details regarding recruitment of patients, information to patients, and plans for follow-up may be improved after input from the users. The planning phase often includes applications for project funding. Active user participation will always improve funding possibilities. An increasing number of funding sources demand user involvement.

During an ongoing research project, there will often be less benefit of user involvement. The patients are not researchers and they are not responsible for the daily tasks such as collecting research data. However, they could be involved in questions such as protocol changes, patient participation, or decisions regarding prolongation of an ongoing study.

When all research data have been collected, the results need to be summarized, discussed, and presented. MG patients may have a role in scientific presentations, especially in the interpretation of consequences for diagnosis and treatment, including new or modified recommendations. User representatives should be involved in the dissemination of the research results to the society in general, including patient interest groups and organizations. This should facilitate and speed up implementation of new research results. Patients may help in the wording of the new information and secure the clinical relevance. They may also know and have access to important information channels and patient networks. The researchers are responsible for the scientific communication of the research results. It is equally important to communicate the results to neurologists who treat MG, and to the patients. User representatives are good partners in this process, sometimes also as active presenters to an audience.

Patient representation can be secured through surveys, but better through direct involvement, sometimes even as coauthors. However, user representative and co-researcher usually represent two different roles. Patient representatives are often required to get funding, and not all of them are truly involved in the research. To get the full value of the representatives, they need to be properly involved. They must be both able and willing to contribute to the project.

Guideline documents for MG treatment and diagnosis are widely read and cited, and their recommendations are usually accepted and implemented. We recommend always to involve MG patients in the work on such documents. Their involvement, especially in the discussion and writing stages, should promote a broad evaluation of all relevant factors before reaching a decision. Users may suggest and support inclusion of additional items for evaluation, for example regarding physical training, diet, sleep, pain control, long-term side-effects, and quality of life.

User involvement increases the chances for research funding. Funding institutions that demand such involvement grade the patient involvement in the same way as other aspects of the application. Our experience is that a standard statement from a MG patient organization confirming their willingness to cooperate and be involved in the planned project has become standard practice. More rarely we see that the users have been involved already and with specification of their input. Good practice implicates that they have contributed to the application and the project plan. The user representatives should be named, and their planned contribution should be described in the same way as for other partners in the project. It is wise to state where the user input will increase quality and relevance, but also where users will not have an active role.

How the user representatives are included in the MG research group may vary. The involvement should depend on interests and qualifications of the representatives, and on the research questions of the group (33). Usually, it is not meaningful for either patients or researchers that they take part in all meetings and in the day-to-day work. However, regular contact is important to secure influence, mutual interest, and interaction (14). Providing information, support and feedback to the user representatives is a key to effective engagement. In selected articles, the user representatives may appear as coauthors as they have contributed in accordance with the Vancouver requirements and have responsibility for the full content of the final article. Typical examples could be guideline documents and policy papers (2). For most articles, a formal acknowledgment of their contribution is appropriate.

Some research groups offer an honorarium to their user representatives. This formalizes the cooperation and secures involvement. It puts this research partner in a special position compared to the rest of the participants, but it may hamper a more informal and flexible cooperation. For some MG patients, such payment represents a token of appreciation and boosts further involvement. Expenses as a user representative or any loss of ordinary income should usually be compensated.

User representatives combine several positions (34). They contribute as co-researchers with direct advice. They use their individual MG experience. They represent their patient organization and network, sometimes including their experiences as representatives in previous research projects. Their ordinary professional education and work comes into play. Finally, they may take the position of the concerned citizen, for example regarding health priorities, gender issues, and ethical aspects.

Guidelines have been developed to govern user involvement in research (12, 34, 35). However, challenges persist. They include lack of support and respect, imbalance of power, and lack of acknowledgment of the patients' true experience. Deviations from the agreed principles in the ongoing work are not uncommon, like other research collaborations. A pitfall to avoid is that user involvement takes too much time and resources, even leading to a reduction in research quality and quantity (36). Frameworks and tools have been suggested to facilitate user involvement as a partnership (12, 34, 35). We discuss most of these tools in this article.

## Choosing Patient Representatives

For MG research projects, patients with an experience of MG and the consequences of muscle weakness should be chosen. MG challenges are specific and complex. The value of user involvement relies on self-experience of MG symptoms and MG impairment. Patients with other disorders, for example muscle or nerve disorders, will not be able to give this specific input. There may be a temptation to recruit patients who are at the same time health professionals. We will advise against such practice as it may blur the patient and outside perspective. On the other hand, higher education and professional experience may lead to a broader participation and a hybrid position of both lay and professional expertise on research, further strengthening the collaboration (31). A patient representative who is not a healthcare professional may bring something new to the project and even help uncover confirmation bias. Such representatives may bring to light new aspects and see the project from an alternative angle. Both researcher and user representative need to reflect on their position in the partnership (37).

MG phenotype varies. It is usually not possible to include both a youngster and an elderly person, one with a mild disease and one who have experienced an MG crisis, or user representatives from all defined MG-subgroups. One or two patients need to cover all aspects. However, for a research group with a special interest in MG crisis, they should involve a patient who have experienced this manifestation. For our research group with an interest in pregnancy and consequences for the child, we have included a young female with children. This ensures the relevance of the patient perspective. For juvenile MG, the perspective of the parents is highly relevant, and guardians can be chosen as user representatives in some projects.

MG patient representatives may be recruited directly by the research group from their patient population. A good alternative is to ask the local or national MG patient organization to find a motivated and able candidate. This should strengthen the responsibilities of all partners and secure interaction with the wider patient community. Some hospitals have user panels that are willing to assist in finding representatives to research projects. However, such representatives should be true MG patients, not just professionals working in an interest organization.

MG patient representatives in a research project may sometimes feel lonely among the group of professional researchers (32). Input from other patients and other user representatives benefits their contribution and increase their motivation. Such input can most importantly come from MG patient networks and organizations, but also from networks of patient representatives for various other neurological and non-neurological disorders.

User representatives will be resourceful, interested in research, and usually well adapted in society. The same is usually true for the active MG researchers. In contrast, MG patients with the highest needs are often those with the least resources; poor socioeconomic conditions, lack of near family and friends, comorbidities, sometimes abuse. Such patients may be disengaged from the medical system. They are not good candidates as user representatives in research projects as they will be unable to contribute properly. However, it is important that the perspective that they represent is included both in the planning and execution of the project, and in the dissemination and implementation of the results.

## Recommendations

All MG research groups should have formal cooperation with user representatives that give regular input to each project. These representatives should be patients who have MG. The focus for the research group should have a strong influence on the choice of user representative. This representative should be involved in the discussions of all relevant questions during the research process. The MG user contribution is especially important in the planning phase of the project, in recruiting MG patients to the project, in the dissemination of results, and for the implementation of the new findings into clinical practice. In applications for research funding, patient representatives should be involved early, and their contribution throughout the project should be specified. Partnership between patients and MG researchers increases research quality and relevance, is motivating for the researchers, and secures support from the society.

## Author Contributions

NG conceived the idea and designed the study, searched the literature, and wrote the first draft of the manuscript. SH contributed in planning of the study, searched the literature, and wrote parts of the manuscript. Both authors contributed to the article and approved the submitted version.

## Conflict of Interest

The authors declare that the research was conducted in the absence of any commercial or financial relationships that could be construed as a potential conflict of interest.

## Publisher's Note

All claims expressed in this article are solely those of the authors and do not necessarily represent those of their affiliated organizations, or those of the publisher, the editors and the reviewers. Any product that may be evaluated in this article, or claim that may be made by its manufacturer, is not guaranteed or endorsed by the publisher.
